# Supplementary material for: Conformation of the nuclear pore in living cells is modulated by transport state
Source: eLife. 2020 Dec 21;9:e60654. doi: 10.7554/eLife.60654 (PMC7752133; doi:10.7554/eLife.60654)
Supplement: Supplementary file 1. — The size of mEGFP deletion describes the number of amino acids deleted from the amino terminus of mEGFP and the net linker size describes the number of amino acids in the linker minus the deletions from the Nup and mEGFP. [file elife-60654-supp1.docx]

**Table S1: Nup-mEGFP Transfected Fusion Proteins**

Size of mEGFP Deletion: Number of amino acids deleted from the amino terminus of mEGFP.

Net Linker Size: Number of amino acids in linker minus the deletions from Nup and mEGFP.

| **Construct Name** | **Nup** | **Position of mEGFP within Nup** | **Size of mEGFP Deletion**  **(AAs)** | **Net Linker Size**  **(AAs)** | **Amino Acid Linker Sequence**  **Nup Sequence in Blue**  **mEGFP Sequence in Green** | **Figures** |
| --- | --- | --- | --- | --- | --- | --- |
| Nup133_mEGFP(-8a)* | Nup133 | Carboxyl-Terminus -2 | 6 | **- 8** | EYYVQGELFT | Fig. 2A;  Fig. 3A |
| Nup133_mEGFP(-8b) | Nup133 | Carboxyl-Terminus -3 | 5 | **- 8** | EYYVQEELFT | Fig. 2A;  Fig. S3A |
| Nup133_mEGFP(-9a) | Nup133 | Carboxyl-Terminus -3 | 6 | **- 9** | EYYVQELFT | Fig. 2A;  Fig. S3B |
| Nup133_mEGFP(-9b) | Nup133 | Carboxyl-Terminus -4 | 5 | **- 9** | EYYVEELFT | Fig. 2A;  Fig. S3C |
| Nup93_mEGFP(-5)** | Nup93 | Carboxyl -Terminus | 5 | **-5** | EVLMNEELFT | Fig. 2B;  Fig. 3B |
| Nup93_mEGFP(-6) | Nup93 | Carboxyl -Terminus | 6 | **-6** | EVLMNELFT | Fig. 2B;  Fig. S3D |
| Nup58_mEGFP(-6) | Nup58 | 412 | 6 | **-6** | RKMFLGELFT | Fig. 2C;  Fig. S3E |
| Nup58_mEGFP(-7) | Nup58 | 410 | 5 | **-7** | RKMFEELFT | Fig. 2C;  Fig. S3F |
| Nup58_mEGFP(-8)*** | Nup58 | 409 | 5 | **-8** | RKMEELFT | Fig. 2C;  Fig. 3C |
| Nup54-mEGFP^494^(0)**** | Nup54 | 494AA + 5AA rigid | 5 | **0** | DIKLVAEAAAEELFT | Fig. 1F;  Fig. 3D |
| Nup54-mEGFP^494^(1) | Nup54 | 494AA + 6AA rigid | 5 | **1** | DIKLVAEAAAKEELFT | Fig. 1F;  Fig. S3G |
| Nup54-mEGFP^494^(2) | Nup54 | 494AA + 7AA rigid | 5 | **2** | DIKLVAEAAAKEEELFT | Fig. 1F;  Fig. S3H |
| Nup54-mEGFP494  (flex0) | Nup54 | 494AA + 5AA flex | 5 | **0** | DIKLVGGGGSEELFT | Fig. 1G |
| Nup54-mEGFP494  (flex1) | Nup54 | 494AA + 6AA flex | 5 | **1** | DIKLVGGGGSSEELFT | Fig. 1G |
| Nup54-mEGFP494  (flex2) | Nup54 | 494AA + 7AA flex | 5 | **2** | DIKLVGGGGSSGEELFT | Fig. 1G |
| Nup54_mEGFP^494^(-4) | Nup54 | 494 | 4 | **-4** | DIKLVGEELFT | Fig. 2D |
| Nup54_mEGFP^494^(-5) | Nup54 | 494 | 5 | **-5** | DIKLVEELFT | Fig. 2D |
| Nup54_mEGFP^494^(-6) | Nup54 | 494 | 6 | **-6** | DIKLVELFT | Fig. 2D |
| Nup54_mEGFP^510^(-4)***** | Nup54 | 510 | 4 | **-4** | GGVFSGEELFT | Fig. 2E;  Fig. 3E |
| Nup54_mEGFP^510^(-5) | Nup54 | 510 | 5 | **-5** | GGVFSEELFT | Fig. 2E |
| Nup54_mEGFP^510^(-6) | Nup54 | 510 | 6 | **-6** | GGVFSELFT | Fig. 2E |

* This construct is the Nup133_mEGFP construct used in the transient transfection starvation experiment in Figure 3.

** This construct is the Nup93_mEGFP construct used in the transient transfection starvation experiment in Figure 3.

*** This construct is the Nup58_mEGFP construct used in the transient transfection starvation experiment in Figure 3.

**** This construct is the Nup54_mEGFP494 construct used in the transient transfection starvation experiment in Figure 3.

**** This construct is the Nup54_mEGFP510 construct used in the transient transfection starvation experiment in Figure 3.
