# Supplementary material for: Conformation of the nuclear pore in living cells is modulated by transport state
Source: eLife. 2020 Dec 21;9:e60654. doi: 10.7554/eLife.60654 (PMC7752133; doi:10.7554/eLife.60654)
Supplement: Supplementary file 2. — The size of mEGFP deletion describes the number of amino acids deleted from the amino terminus of mEGFP and the net linker size describes the number of amino acids in the linker minus the deletions from the Nup and mEGFP. [file elife-60654-supp2.docx]

**Table S2: Crispr Nup-mEGFP Cell Lines**

| **Construct Name** | **Nup** | **Position of mEGFP within Nup** | **Size of mEGFP Deletion**  **(AAs)** | **Net Linker Size**  **(AAs)** | **Amino Acid Linker Sequence**  **Nup Sequence in Blue**  **mEGFP Sequence in Green** | **Figures** |
| --- | --- | --- | --- | --- | --- | --- |
| Nup133_mEGFP(-9)^†^ | Nup133 | Carboxyl-Terminus -3 | 6 | **- 9** | EYYVQELFT | Fig. 4C,E,G,I,K; Fig. 5 |
| Nup133_mEGFP(-8) | Nup133 | Carboxyl-Terminus -2 | 6 | **- 8** | EYYVQGELFT | Fig. 4C; Fig. S5A |
| Nup54-mEGFP^494^(0)^††^ | Nup54 | 494AA + 5AA rigid | 5 | **0** | DIKLVAEAAAEELFT | Fig. 4D,F,H,J,L; Fig. 5 |
| Nup54-mEGFP^494^(1) | Nup54 | 494AA + 6AA rigid | 5 | **1** | DIKLVAEAAAKEELFT | Fig. 4D; Fig. S5B |
| Nup54-mEGFP^494^(2) | Nup54 | 494AA + 7AA rigid | 5 | **2** | DIKLVAEAAAKEEELFT | Fig. 4D; Fig. S5C |

^†^ This cell line is the Nup133_mEGFP cell line used for the experiments to reduce transport in Figure 4 and the experiments to manipulate transport factors in Figure 5.

^††^ This cell line is the Nup54_mEGFP cell line used for experiments to reduce transport in Figure 4 and the experiments to manipulate transport factors in Figure 5.
